# Dataset of allele and genotype frequencies of the three functionally significant polymorphisms of the MMP genes in Russian patients with primary open-angle glaucoma, essential hypertension and peptic ulcer

**DOI:** 10.1016/j.dib.2020.106004

**Published:** 2020-07-08

**Authors:** Oksana Minyaylo, Dina Starikova, Maria Moskalenko, Irina Ponomarenko, Evgeny Reshetnikov, Volodymyr Dvornyk, Mikhail Churnosov

**Affiliations:** a Department of Medical Biological Disciplines, Belgorod State University, Belgorod 308015, Russia; b Department of Life Sciences, College of Science and General Studies, Alfaisal University, Riyadh 11533, Saudi Arabia

**Keywords:** Single nucleotide polymorphism, Primary open-angle glaucoma, Essential hypertension, Peptic ulcer, *MMP*

## Abstract

Data on the allele and genotype frequencies of the three functionally significant single nucleotide polymorphisms (SNPs) of the matrix metalloproteinases (*MMP*) genes (rs1799750 *MMP1*, rs3918242 and rs17576 *MMP9*) in Russian patients with primary open-angle glaucoma (POAG), essential hypertension (EH) and peptic ulcer (PU) are presented. Association studies identified these SNPs as possible significant markers associated with many multifactorial disorders, including POAG, EH, and PU. The frequencies of alleles and genotypes of the three SNPs in Russian patients with POAG, EH, and PU were presented separately for the entire study sample, females, and males, respectively. The data can be used as a reference for the Russian population.

**Specifications Table**

**Table:** 

**Subject**	*Biology*
**Specific subject area**	*Genetics*
**Type of data**	*Table and figure*
**How data were acquired**	*MALDI/TOF mass spectrometry using the Sequenom MassARRAY 4.0 platform (Agena Bioscience™)*
**Data format**	*Raw and analyzed data*
**Parameters for data collection**	*Whole blood (5* *ml) was drawn to a plastic vial (Vacutainer^Ⓡ^) containing 0.5* *M EDTA (рН=8.0). Genomic DNA was isolated by the standard phenol-chloroform method. DNA samples were first checked for quality (concentration 10–15* *ng/mL, purity А260/А280=1.7–2.0) and then used for genotyping. About 5% of blind replicate samples were used for genotyping quality control; the repeatability test indicated a 100% concordance rate.*
**Description of data collection**	*The quality of isolated DNA was checked by the Nanodrop-2000 spectrophotometer. Genotyping was performed on the Sequenom MassARRAY^Ⓡ^ iPLEX platform using the MALDI-TOF (matrix-assisted laser desorption/ ionization time-of-flight) mass spectrometry. Assay Design Suite 1.0 was used to design a multiplex genotyping assay* (http://agenabio.com/assay-design-suite-10-software)*.*
**Data source location**	*Belgorod, Russia*
**Data accessibility**	*The data is available with this article*

**Value of the data**

• The frequencies of alleles and genotypes of rs1799750 *MMP1*, rs3918242 and rs17576 *MMP9* in Russian patients with POAG, EH, and PU are presented separately for the entire cohort, male and female participants.
• The polymorphisms rs1799750 *MMP1*, rs3918242 and rs17576 *MMP9* may be associated with POAG, EH, and PU.
• The data on the allele and genotype frequencies of the *MMP* genes can be used for meta-analyses of genetic studies on POAG, EH, and PU.
• The presented data of the *MMP* genes polymorphisms can serve as a reference for population and genetic association studies of the common disorders.

## 1 Data description

The dataset contains the raw data (supplementary Table), frequencies of alleles and genotypes (Table 1) for three SNPs of two *MMP* genes (rs1799750 *MMP1*, rs3918242 and rs17576 *MMP9*) in Russian patients diagnosed with POAG, EH, and PU. These polymorphisms were previously reported for their association with POAG, EH, and PU (Table 2) [1], [2], [3], [4], [5], [6], [7], [8], [9], [10], [11], [12], [13], [14], [15], [16], [17], [18], [19], [20], [21], [22], [23], [24], [25], [26], [27], [28], [29], [30], [31], [32], [33], [34], [35], [36], [37], [38], [39], [40], [41], [42], [43], [44], [45]. The studied SNPs manifest the regulatory potential (Table 3), which is evidenced by several eQTLs (Table 4) and splicing QTLs (Table 5). The allele and genotype frequencies are provided separately for the whole study cohort, females, and males, respectively. No significant differences in the frequencies of alleles and genotypes were found between the male and female participants for each of the studied diseases.

**Table 1:** The frequencies of alleles and genotypes for SNPs rs1799750 *MMP1*, rs3918242 and rs17576 *MMP9* in Russian patients with POАG, EH, and PU.

Diseases	SNP genotype or allele	All		Male			Female	
		n	frequency	n	frequency		n	frequency
	rs1799750							
POAG	1G1G	152	0.2836	73	0.2968		79	0.2724
	1G2G	267	0.4981	131	0.5325		136	0.4690
	2G2G	117	0.2183	42	0.1707		75	0.2586
	1G	571	0.5327	277	0.5630		294	0.5069
	2G	501	0.4673	215	0.4370		286	0.4931
	rs3918242							
	СС	385	0.7183	175	0.7114	210		0.7241
	СТ	133	0.2482	65	0.2642	68		0.2345
	ТТ	18	0.0335	6	0.0244	12		0.0414
	С	903	0.8424	415	0.8435	488		0.8414
	Т	169	0.1576	77	0.1565	92		0.1586
	rs17576							
	AA	205	0.3825	110	0.4472	95		0.3276
	GA	260	0.4851	108	0.4390	152		0.5241
	GG	71	0.1324	28	0.1138	43		0.1483
	A	670	0.6250	328	0.6667	342		0.5897
	G	402	0.3750	164	0.3333	238		0.4103
EH	rs1799750							
	1G1G	169	0.2721	65	0.2481	104		0.2897
	1G2G	309	0.4976	140	0.5334	169		0.4707
	2G2G	143	0.2303	57	0.2175	86		0.2396
	1G	647	0.5209	270	0.5153	377		0.5251
	2G	595	0.4791	254	0.4847	341		0.4749
	rs3918242							
	СС	444	0.7150	189	0.7214	255		0.7103
	СТ	149	0.2399	64	0.2443	85		0.2368
	ТТ	28	0.0451	9	0.0343	19		0.0529
	С	1037	0.8349	442	0.8435	595		0.8287
	Т	205	0.1651	82	0.1565	123		0.1713
	rs17576							
	AA	229	0.3688	100	0.3817	129		0.3593
	GA	311	0.5008	131	0.5000	180		0.5014
	GG	81	0.1304	31	0.1183	50		0.1393
	A	769	0.6192	331	0.6317	438		0.6101
	G	473	0.3808	193	0.3683	280		0.3899
РU	rs1799750							
	1G1G	121	0.3033	45	0.2394	76		0.3602
	1G2G	195	0.4887	98	0.5212	97		0.4597
	2G2G	83	0.2080	45	0.2394	38		0.1801
	1G	437	0.5476	188	0.5000	249		0.5901
	2G	361	0.4524	188	0.5000	173		0.4099
	rs3918242							
	СС	277	0.6942	129	0.6862	148		0.7014
	СТ	115	0.2883	58	0.3085	57		0.2701
	ТТ	7	0.0175	1	0.0053	6		0.0285
	С	669	0.8383	316	0.8404	353		0.8365
	Т	129	0.1617	60	0.1596	69		0.1635
	rs17576							
	AA	142	0.3559	69	0.3670	73		0.3460
	GA	184	0.4612	83	0.4415	101		0.4787
	GG	73	0.1829	36	0.1915	37		0.1753
	A	468	0.5865	221	0.5878	247		0.5853
	G	330	0.4135	155	0.4122	175		0.4147

Abbreviations: POAG - primary open-angle glaucoma, EH - essential hypertension, PU - peptic ulcer.

**Table 2:** The literature data about associations of the studied polymorphisms of the *ММР* genes with POАG, PU and some digestive diseases (gastric and esophageal cancer), EH and IS with EH.

SNP	Gene	Number of publications in PubMed/PubMed Central	Phenotype	Association (significance) (associated allele)	Reference
rs1799750	*MMP1*	70/119	POAG	OR = 1.64, р = 0.01	[1]
			POAG	OR = 1.64, *p* = 0.002	[2]
			POAG	OR = 1.34, р = 0,017 (2 G)	[3]
			POAG	OR = 2.04, р<0,001 (2 G)	[4]
			POAG	OR = 1.35, *p* = 0.017 (2 G)	[5]
			POAG	*p*>0.05	[6]
			peptic ulcer	OR = 3.46, *p* = 0.03 (1 G/1 G)	[7]
			gastric cancer	OR = 3.34, р = 0.016 (2 G/2 G)	[8]
			gastric cancer	OR = 1.05, р = 0.013 (2 G)	[9]
			IS with hypertension	OR = 1.54, *p* = 0.005 (2 G)	[10]
			IS with hypertension	OR>1; *p*<0.05 (2 G)	[11]
			IS with hypertension	*p*>0.05	[12], [13], [14], [15], [16], [17]
			essential hypertension in men	OR = 2.58; р = 0.04 (together with rs11568818, rs1320632, rs11225395)	[18]
			essential hypertension in women	*p*>0.05	[19]
rs3918242	*MMP9*	106/127	POAG	OR = 1.63; р = 0.002 (Т)	[20]
			POAG	OR = 1.55, *p* = 0.012 (T)	[5]
			POAG	OR = 1.46, *p* = 0.032 (CT+TT)	[21]
			POAG	*p*>0.05	[1,4,22]
			peptic ulcer	*p*>0.05	[7]
			gastric cancer	OR = 2.60; р<0.05 (together with rs17576 and rs17577)	[23]
			esophageal cancer	OR = 2.71; р = 0.02 (СС)	[24]
			gastric cancer	*p*>0.05	[25]
			IS with hypertension	OR = 2,76; р = 0.003 (ТT)	[26]
			IS with hypertension	OR = 1,73; р<0,05 (Т)	[27]
			IS with hypertension	OR = 2,20; р<0,05 (ТТ)	[28]
			IS with hypertension	OR = 2,08; р = 0,016 (Т)	[29]
			IS with hypertension	OR<1; р = 0.001 (CC)	[30]
			IS with hypertension	OR>1; р = 0.009 (T)	[31]
			IS with hypertension	OR = 5,53; р = 0,001 (СС)	[32]
			IS with hypertension	OR = 1.43; р = 0.001 (Т)	[33]
			IS with hypertension	OR = 5.47; р<0.05 (TТ)	[34]
			IS with hypertension	OR = 1.27; р = 0.01 (T)	[12]
			IS with hypertension	*p*>0.05	[35]
			essential hypertension	OR = 1.30; р = 0.002	[36]
			isolated systolic hypertension	OR>1; р = 0,009 (T)	[37]
			left ventricular hypertrophy in hypertensive patients	OR>1; р = 0,0015 (together with rs2234681 and rs17576)	[38]
			hypertension of pregnancy	OR<1; р = 0,007 (СС)	[39]
			essential hypertension in children	OR>1; р<0.05 (ТТ)	[40]
			essential hypertension	*p*>0.05	[41]
rs17576	*MMP9*	78/97	POAG	OR = 1.96; р = 0.0005 (AG)	[20]
			POAG	OR = 0.66; *p* = 0.03 (A)	[21]
			POAG	OR = 1.53; *p* = 0.034 (GG)	[42]
			POAG in men	OR = 0.56; р = 0,003 (together with rs2250889)	[43]
			POAG	OR = 2.34; *p* = 0.01 (GG)	[4]
			POAG	*p*>0.05	[6]
			peptic ulcer	OR = 0.49; *p* = 0.007 (AA)	[7]
			gastric cancer	OR = 4.34; р<0.05 (Q)	[23]
			gastric cancer	*p*>0.05	[44]
			IS with hypertension	OR = 0,91; р = 0,04 (GG)	[45]
			IS with hypertension	*p*>0.05	[15,26,29,30,31]
			left ventricular hypertrophy in hypertensive patients	OR>1; р = 0,0015 (together with rs2234681 and rs3918242)	[38]
			essential hypertension	OR>1; р<0.05 (АА)	[41]
			isolated systolic hypertension	*p*>0.05	[37]

Abbreviations: POAG - primary open-angle glaucoma, EH - essential hypertension, IS - ischemic stroke, PU - peptic ulcer.

**Table 3:** Regulatory effects of the 3 SNPs of the *MMP* genes (HaploReg, v4.1, update 05.11.2015) (https://pubs.broadinstitute.org/mammals/haploreg/haploreg.php).

**Table 4:** The cis-eQTL values of the 3 SNPs of the *MMP* genes. (according to Genotype-Tissue Expression (GTEx) (http://www.gtexportal.org/)).

Chr	SNP	Reference allele	Alternative allele	Gene expression	Effect Size (β)	P-Value	Tissue
11	rs1799750	TC	T	*MMP1*	−0.66	9.6E-84	Cells - Cultured fibroblasts
				*MMP1*	−0.52	1.3E-25	Thyroid
				*MMP1*	−0.42	1.9E-25	Lung
				*MMP1*	−0.58	5.8E-23	Heart - Atrial Appendage
				*MMP1*	−0.45	0.0000000000000000017	Adipose - Visceral (Omentum)
				*MMP1*	−0.46	0.0000000000000066	Heart - Left Ventricle
				*MMP1*	−0.36	0.000000000022	Nerve - Tibial
				*MMP1*	−0.32	0.000000013	Esophagus - Muscularis
				*MMP1*	−0.35	0.000000015	Artery - Aorta
				*MMP1*	−0.28	0.000000024	Adipose - Subcutaneous
				*MMP1*	−0.22	0.00000038	Artery - Tibial
				*MMP10*	−0.19	0.0000025	Lung
				*MMP1*	−0.3	0.0000028	Esophagus - Gastroesophageal Junction
				*MMP1*	−0.28	0.0000075	Breast - Mammary Tissue
				*WTAPP1*	−0.15	0.00004	Testis
20	rs3918242	C	T	*SLC12A5*	0.61	0.0000000000000000059	Lung
				*SLC12A5*	0.8	0.0000000000000016	Adipose - Visceral (Omentum)
				*SLC12A5*	0.6	0.000000000045	Adipose - Subcutaneous
				*SLC12A5*	0.69	0.000000045	Breast - Mammary Tissue
				*SLC12A5*	0.63	0.000000082	Artery - Aorta
				*SLC12A5*	0.78	0.00000018	Spleen
				*SLC12A5*	−0.61	0.00000019	Adrenal Gland
				*SNX21*	0.21	0.00000099	Muscle - Skeletal
				*SLC12A5*	0.45	0.0000029	Thyroid
				*SLC12A5*	0.43	0.0000037	Nerve - Tibial
				*SLC12A5*	0.43	0.0000038	Uterus
				*SLC12A5*	0.41	0.0000053	Skin - Sun Exposed (Lower leg)
				*SLC12A5*	0.48	0.00001	Skin - Not Sun Exposed (Suprapubic)
				*PLTP*	−0.25	0.000028	Nerve - Tibial
20	rs17576	A	G	*SLC12A5*	−0.57	0.0000000000043	Adrenal Gland
				*PLTP*	−0.26	0.00000000033	Lung
				*PLTP*	−0.32	0.000000026	Heart - Left Ventricle
				*PLTP*	−0.24	0.000000034	Nerve - Tibial
				*PLTP*	−0.2	0.00000021	Adipose - Subcutaneous
				*NEURL2*	−0.3	0.00000026	Adipose - Visceral (Omentum)
				*PLTP*	−0.2	0.00000046	Thyroid
				*PLTP*	−0.19	0.00000055	Artery - Tibial
				*PLTP*	−0.37	0.00000066	Adrenal Gland
				*NEURL2*	−0.24	0.0000015	Adipose - Subcutaneous
				*PLTP*	−0.25	0.000002	Artery - Aorta
				*NEURL2*	−0.31	0.0000021	Artery - Aorta
				*PLTP*	−0.18	0.0000024	Adipose - Visceral (Omentum)
				*PLTP*	−0.3	0.0000025	Colon - Sigmoid
				*PLTP*	−0.22	0.0000033	Brain - Frontal Cortex (BA9)
				*PCIF1*	0.35	0.0000041	Adrenal Gland
				*ZSWIM1*	−0.26	0.0000068	Adipose - Visceral (Omentum)
				*RP3–337O18.9*	−0.22	0.0000076	Lung
				*PLTP*	−0.32	0.0000091	Pituitary
				*SNX21*	0.14	0.000012	Muscle - Skeletal
				*RP3–337O18.9*	−0.2	0.000028	Adipose - Subcutaneous
				*NEURL2*	−0.22	0.000036	Lung
				*NEURL2*	−0.22	0.000073	Thyroid

**Table 5:** The sQTL values of the 3 SNPs of the *MMP* genes. (according to Genotype-Tissue Expression (GTEx) (http://www.gtexportal.org/)).

Chr	SNP	Reference allele	Alternative allele	Gene Symbol	Intron Id	Effect Size (β)	P-Value	Tissue
11	rs1799750	TC	T	*WTAPP1*	102,832,906:102,833,452:clu_16,168	−0.51	0.000000000065	Testis
20	rs3918242	C	T	*CD40*	46,126,741:46,128,138:clu_33,045	0.5	0.0000000029	Thyroid
				*CD40*	46,126,741:46,128,138:clu_32,508	0.45	0.0000000067	Lung
				*CD40*	46,126,741:46,128,138:clu_32,508	0.45	0.0000000067	Lung
				*SLC12A5*	46,021,886:46,023,369:clu_29,529	0.79	0.0000000098	Pituitary
				*CD40*	46,126,741:46,128,138:clu_27,442	0.49	0.00000041	Artery - Aorta
				*ACOT8*	45,841,956:45,844,263:clu_24,540	0.58	0.0000011	Heart - Left Ventricle
				*CD40*	46,126,741:46,128,138:clu_22,055	0.74	0.0000015	Cells - EBV-transformed lymphocytes
				*ACOT8*	45,841,956:45,844,263:clu_27,123	0.49	0.0000041	Heart - Atrial Appendage
20	rs17576	A	G	*SLC12A5*	46,021,886:46,023,369:clu_29,529	0.63	0.0000000000093	Pituitary
				*SLC12A5*	46,023,071:46,023,369:clu_24,852	−0.45	0.00000033	Brain - Cortex
				*SLC12A5*	46,021,886:46,023,369:clu_26,648	0.46	0.000002	Brain - Cerebellum
				*SLC12A5*	46,021,886:46,023,369:clu_53,353	0.38	0.000011	Testis

## 2 Experimental design, materials, and methods

### 2.1 Study subjects

The study cohort consisted of 1556 Russian participants, including 536 patients diagnosed with POAG (290 females and 246 males), 621 patients with EH (359 females and 262 males), and 399 patients with PU (211 females and 188 males). The study participants were clinically examined at the Department of Eye Microsurgery (patients with POAG), Department of Cardiology (patients with EH), and Department of Gastroenterology (patients with PU) of St. Iasaf Belgorod Regional Clinical Hospital. All participants were self-reported unrelated Russians born in Central Russia [46]. The study was approved by the Regional Ethics Committee of Belgorod State University. All participants signed an informed consent prior to the enrolment to this study.

### 2.2 DNA analysis

Phlebotomy was performed by a certified nurse. Blood (5 ml) was drawn from the ulnar vein to a plastic vial (Vacutainer^Ⓡ^) with 0.5 M EDTA (рН = 8.0). Total genomic DNA was isolated from the buffy coat by the standard phenol-chloroform protocol [47] and then checked for quality using Nanodrop 2000 spectrophotometer (Thermo Scientific, Inc.). Only samples with А260/А280 = 1.7–2.0 were used for the analysis. The isolated DNA was stored at −80°С.

Three SNPs of the *MMP* genes (rs1799750 *MMP1*, rs3918242 and rs17576 *MMP9*) were selected for the analysis. The following selection criteria were applied [48,49]: 1) Previously reported associations with POAG, EH and PU (Table 2), 2) Regulatory potential (regSNP) (Table 3), 3) Effect on gene expression (eSNP) (Table 4), 4) Splicing QTLs (sSNP) (Table 5), and 5) MAF > 5%.

The selected loci were associated with POAG, EH and PU in previously published candidate gene association studies (Table 2) and have functional significance: significant regulatory potential (Table 3) (determined using the online tools HaploReg, v4.1 update 05.11.2015, https://pubs.broadinstitute.org/mammals/haploreg/haploreg.php), influence gene expression level (Table 4) and involved in splicing QTLs (Table 5) (determined using the GTExportal data, http://www.gtexportal.org/).

The DNA samples used for the analysis had concentration 10–15 ng/ml. A single well iPLEX SNP genotyping assay was designed using the Assay Design Suite 1.0 (http://agenabio.com/assay-design-suite-10-software). For this purpose, three SNPs of interest were retrieved from dbSNP of NCBI and imported into the software according to their IDs. DNA genotyping was performed on the MALDI‐TOF mass spectrometry iPLEX platform (Agena Bioscience Inc, San Diego, CA).

For quality control of genotyping, 5% of blind replicate samples were included. The concordance for replicate samples was 100%.

### 2.3 Statistical analysis

The studied SNPs were checked for their correspondence to the Hardy-Weinberg equilibrium (HWE) using the chi-square test. The frequencies of alleles and genotypes were analyzed for possible differences between the females and males in the study sample using the Kruskall-Wallis test.

## Declaration of Competing Interest

The authors have no known competing financial interests or personal relationships that might have, or could be perceived to have influenced the results reported in this article.

## Appendix Supplementary materials

Image, application 1

Image, application 2
